# Explicating the concept of epistemic rationality

**DOI:** 10.1007/s11229-020-03011-5

**Published:** 2021-02-20

**Authors:** Anna-Maria A. Eder

**Affiliations:** University of Cologne, Cologne, Germany

**Keywords:** Conceptual engineering, Conceptual ethics, Epistemic rationality, Epistemological methodology, Explication, Rudolf Carnap, Epistemic normativity, Epistemic pluralism

## Abstract

A characterization of epistemic rationality, or epistemic justification, is typically taken to require a process of conceptual clarification, and is seen as comprising the core of a theory of (epistemic) rationality. I propose to explicate the concept of rationality. It is essential, I argue, that the normativity of rationality, and the purpose, or goal, for which the particular theory of rationality is being proposed, is taken into account when explicating the concept of rationality. My position thus amounts to an instrumentalist position about theories of epistemic rationality. Since there are different purposes, or goals, for which theories of rationality are proposed, the method of explication leaves room for different characterizations of rationality. I focus on two such (kinds of) purposes: first, the purpose of guiding the formation (or maintenance) of doxastic states and, second, the purpose of assessing (the formation or maintenance of) doxastic states. I conclude by outlining a pluralistic picture concerning rationality.

## Introduction

### General setting and main aim

One of the primary tasks in epistemology is to provide a characterization of epistemic rationality, or epistemic justification.^1^ As understood here, a characterization of rationality tells us when it is rational for an agent to believe a proposition. Such a characterization is typically taken to be the outcome of a process of conceptual clarification, and as comprising the core of a theory of rationality. The method of conceptual analysis is a traditional method of conceptual clarification in philosophy.^2^ After years of analysis of the concept of (epistemic) rationality, however, there is still fundamental disagreement about the right characterization. In this paper, I propose to utilize an alternative method of conceptual clarification: the method of *explication*.^3^

Rather than suggesting specific characterizations of rationality or specific explications of the concept of rationality, or discussing methods of conceptual clarification in general, I aim to make a contribution to the meta-epistemological foundations of theories of rationality in general. In particular:

**Main Aim**: My main aim is to outline the method of explication as it should be applied to the concept of epistemic rationality.

I focus on features of the method that are characteristic for it when it is applied to the concept of epistemic rationality, and outline the method in a way that leaves room for several specific explications of the concept of rationality. My outline amounts to a first step towards specific explication(s) of the concept.^4^$$^{,}$$^5^

The standard method of explication, developed by Carnap (1950/1962), has not been sufficiently specified to enable us to use it to clarify the concept of epistemic rationality. I do not seek any further specification of the general method of explication; rather, I seek to specify the method of explication in so far as it might be applied to the concept of epistemic rationality. It is essential, I argue, that the normativity of rationality and the intended purpose, or goal, of the theory of rationality be taken into account when explicating the concept of rationality.^6^ What I propose amounts to an instrumentalist position about theories of rationality: it is an instrumentalist position that concerns how to characterize rationality, and how to clarify the concept of rationality.^7^ Given the differences between such purposes, the method of explication that I put forward allows for different characterizations of rationality and, thus, for epistemic pluralism concerning rationality.

### Plan

To achieve my main aim, I proceed as follows: I start (in Sect. 2) by introducing methods of conceptual clarification: I present the method of (traditional) conceptual analysis and discuss its shortcomings concerning the concept of rationality, and then propose the method of explication as an alternative. After comparing the methods briefly, I focus on the latter (Sect. 3). The standard conception of explication, however, is not sufficiently specific to enable us to employ it to clarify the concept of rationality. Thus I emphasize that the normativity of rationality and the purposes for which theories of rationality are proposed are relevant for the explication of the concept. In this paper, I focus on two such (kinds of) purposes: first, the purpose of guiding the formation (or maintenance) of doxastic states and, second, the purpose of assessing (the formation or maintenance of) doxastic states. Based on my outline of the method of explication, I then sketch a pluralistic picture concerning rationality by focusing in more detail on those purposes (Sect. 4). I conclude by summarizing my findings (Sect. 5).

## Conceptual clarification in epistemology

### Traditional conceptual analysis

Different conceptions of the method of conceptual analysis are at work in philosophy. Here I focus on a conception coined by Strawson (1963, 1992), which I refer to as *traditional conceptual analysis* (or, for brevity, ‘conceptual analysis’). By contrasting this with explication, we shall get a better grip on the method of explication itself. I leave for another occasion a comparison between the method of explication and alternative methods of conceptual clarification.^8^ My main points can be made sufficiently clear by contrasting the method of explication with conceptual analysis as here understood.

The method of (traditional) conceptual analysis is a method of clarifying a concept, termed the ‘analysandum’. Conceptual analyses start with an *informal clarification* of the analysandum, and aim to give a *definition* of it by way of providing a concept deemed synonymous, the *analysans*. (For the sake of simplicity I use the term ‘analysandum’ to refer to concepts as well as to the respective terms. It will be clear from the context what is meant by ‘analysandum’.) The analysandum is then the definiendum of the definition, and the definition is considered to be analytically true. Depending on the specific account, the analysans is supposed to satisfy further requirements besides the synonymy condition.

Conceptual analyses, as understood here, are meant to be carried out on concepts that are sufficiently exact for philosophical purposes and, thus, not defective for such purposes. Strawson, for example, seems to assume that philosophical problems are stated in ordinary language, and that philosophical concepts that are expressed in ordinary language are sufficiently exact for philosophical purposes. (This is not to claim that it is required that the analysanda are exact simpliciter.) According to Strawson, it is characteristic for conceptual analyses that the analysandum is drawn from ordinary language, and he thinks that philosophers are sufficiently competent language users to know how to use the respective concepts that might bring about confusion (Strawson 1963, 1992, p. 7). The result of a traditional conceptual analysis, that is, the definition, is a *description* that reflects how the analysandum is appropriately used in ordinary language (Strawson 1963; see also Justus 2012). In what follows, I discuss two shortcomings of conceptual analysis when it comes to clarifying the concept of rationality.

The first shortcoming is that conceptual analysis is intended to clarify concepts that are exact enough. Yet we have reason to believe that the concept of rationality is not exact enough for epistemological purposes; it is inexact, and indeed defective for these purposes. Within epistemology, there is much disagreement concerning the right characterization of rationality and the right conceptual analysis of the concept of rationality. The evidentialism/reliabilism and internalism/externalism debates concerning rationality, or justification, show the extent of the disagreement very clearly. Moreover, ascriptions of rationality often differ considerably concerning specific cases, and *no* reason has been given for assuming that there is one *exact* concept.^9^ These comprehensive disagreements could hardly be explained if the concept were exact enough and not defective for epistemological purposes. I take the disagreements to be a strong indicator for some form of inexactness (e.g., ambivalence, incoherence, or vagueness in all or some contexts)^10^ or defectiveness (for our epistemological purposes).^11^

Exactly what kind of inexactness is involved will not be essential for my outline of the method of explication as it is applied to the concept of rationality. What is relevant here is that the concept of rationality is inexact for epistemology; it has some defects irrespective of what these might be. The reasons that I mentioned for believing that the concept of rationality is not exact (and is therefore defective) are not decisive here. I do not claim that all forms of disagreement arise from the inexactness of the concepts in question, nor do I claim that all inexactness results in disagreement. Nevertheless, in the present context, this disagreement suffices to motivate an investigation into alternative methods of conceptual clarification. Before we do so, however, let us discuss the second shortcoming of conceptual analysis as regards the concept of rationality.

The second shortcoming is that conceptual analysis is meant to clarify concepts of ordinary language. This characteristic feature is lacking in the case of the concept of epistemic rationality. Although in *ordinary language* people may talk about *moral* or *ethical* rationality, or justification, the concepts of epistemic rationality and the terms used for it are not common in ordinary language. In line with this, Hawthorne and Stanley remark that “‘justified belief’ is a phrase from philosophy classrooms” (2008, p. 573).^12^

So what is a suitable alternative method for clarifying the concept of rationality? It is time to grant the method of explication its proper place in epistemology and take it seriously as an alternative to conceptual analysis.^13^

### Explication

I introduce the method of explication as proposed and developed by Carnap (1950/1962, 1963, 1988), endorsed and slightly specified by Quine (1960/2013: §53), and adopted by Hempel (1950, 1952: Ch. I).^14^ An *explication* is a method of clarifying a concept. It is characteristic of explications that they replace the concept that is not exact enough (for its purpose), the *explicandum*, by a (more) exact concept, the *explicatum*. The explicatum is supposed to be *similar in use* to the explicandum.^15^ For the sake of simplicity I follow Carnap in using the term ‘explicandum’ to refer to concepts as well as to the respective terms, and the same holds for ‘explicatum’ (Carnap 1950/1962). It will be clear from the context what is meant by ‘explicandum’ and ‘explicatum.’

The method as understood here is a method of *conceptual engineering*. It *engineers* and introduces *new* concepts.^16^ Since the new concept is not arbitrarily chosen, and has to satisfy specific purposes, the method also counts as a method of *conceptual ethics*. As such, the method concerns the question of which concepts we should use given specific purposes or goals.^17^ My investigation should be of value to those who are sympathetic to conceptual engineering and conceptual ethics, even if they are hesitant to accept that the concept of rationality is not exact enough for epistemological purposes or want to insist that the concept of (epistemic) rationality is a concept of ordinary language.

The method starts with an “informal *clarification*” of the explicandum.^18^ This first step is required to assure ourselves that we have identified the explicandum and, for example, to disambiguate the respective term (Carnap 1963; see also Cordes and Siegwart 2018; Brun 2020; Olsson 2015, 2017).

The method ends by stipulating exact rules of use for the explicatum. These rules of use *do not describe* how the explicandum is properly used in ordinary language, but rather *stipulate* how to use the explicatum.^19^ The stipulation should be considered a piece of advice that is useful given specific purposes. Being simply pieces of advice, they can of course be criticized: they may be adequate or inadequate, and whether they are adequate depends on whether they are useful for a given purpose. Carnap says that “whenever greater precision in communication is *desired*, it will be *advisible to use* the explicatum instead of the explicandum” (Carnap 1963, p. 935; my emphasis). Although explications stipulate explicata, they need not provide final decisions. They provide advice in the form of rules of use. In the respect that is relevant here, I think that such rules are similar to the wordings of the law, which are also stipulated and yet nevertheless might be improved upon in the course of discussions. Similarly, explication might also be improved, perhaps as a result of a discourse. Furthermore, the rules of use may be given in the form of an (explicit) definition, but can also be given in some other form (Carnap 1950/1962: 3, 7).^20^ If a definition specifies the rules of use, the explicatum of the explication is the definiendum of the definition (see also Brun 2020).

In order to be an adequate explication, the explicatum has to meet the following requirements to a “sufficient degree”: (i) it is “*similar to the explicandum*”, (ii) its characterization is “given in an *exact* form”, (iii) it is *fruitful*, and (iv) “as *simple* as possible” (Carnap 1950/1962, p. 7). Carnap does not specify when these requirements are met to a sufficient degree—something which commentators have had cause to complain about.^21^ However, I do not consider this to be a shortcoming of Carnap’s account of explication; on the contrary, in both Carnap’s and my own view, “a language, whether natural or artificial, is an instrument that may be replaced or modified according to our needs, like any other instrument” (Carnap 1963, p. 938). In line with this general view on language, it is only natural to say that whether the requirements on explicata are met should depend on the specific purposes that are pursued by specific explications.^22^ This is in line with Quine (1960/2013: §53), who also emphasizes the relevance of the purpose of an explication.^23^ I think that it would be asking too much from Carnap and proponents of general accounts of explication to specify the purposes for any (specific) explication. In what follows I discuss the requirements on explicata in more detail.

#### Similarity requirement

The informal clarification of the explicandum, which corresponds to the first step of an explication, is meant to help to establish that the explicatum is similar (in use) to the explicandum, and so, in turn, to ensure that the explicandum is not replaced by some arbitrarily chosen exact explicatum. Note that Carnap does not require a maximally possible similarity between the explicatum and the explicandum. Good reasons for not requiring it arise, for example, when a higher degree of fruitfulness goes along with a lower degree of similarity (Carnap 1950/1962, p. 5).^24^ Carnap considers the fruitfulness requirement to be more important than the similarity requirement (Carnap 1950/1962, pp. 5–6). However, I follow Quine (1960/2013, p. 241) in requiring that if the suggested explicatum does not display a certain minimal similarity to the explicandum, it does not count as an explication. It is merely an elimination of an old concept followed by the introduction of a new but unrelated concept. I follow Quine in this regard because I would like to delimit explications from mere stipulative introductions of new concepts. The requirement of minimal similarity serves this purpose. Thus according to the picture advanced here, explications are flanked by conceptual analysis on the one side and stipulative introductions of new concepts on the other. The similarity requirement is crucial for the discussion here. In Sect. 3.1, I will discuss minimal similarity conditions on explicata concerning the concept of rationality. This is to make sure that utterly different explicata do not replace the concept of rationality.

#### Exactness requirement

It is implicit in Carnap’s work that the exactness has to be achieved within a formal framework, as Reck (2012: Part 1) rightly notes. However, in some areas in philosophy, seeking formal standards of exactness might be asking too much.^25^ As an alternative, which I hope to be attractive in non-formal philosophical investigations, I suggest that the explicandum be introduced into a restricted part of natural language that mimics, at least partially, mathematically precise languages. For this purpose, philosophers need to specify which concepts they accept as sufficiently clear. In such a language, the quantifiers and connectives should be used in close similarity to how they are used in formal disciplines. I also think that we should require that not only the explicatum but also the terms used to specify the rules of use for the explicatum should be more precise than the explicandum. This gives a good understanding of what is meant by exact rules for the explicatum. I do not commit myself to any additional requirements concerning how much exactness is required for explications. Following Carnap (1963), the level of exactness that is required depends on the purpose of the theory in question, and might differ from explication to explication.

#### Fruitfulness requirement

As mentioned before, Carnap considers the fruitfulness requirement to be more important than the similarity requirement (Carnap 1950/1962, pp. 5–6). For him, an explication is meant to contribute to establishing exact scientific theories by replacing inexact concepts by exact ones that are “useful for the formulation of many universal statements (empirical laws in the case of a nonlogical concept, logical theorems in the case of a logical concept)” (Carnap 1950/1962, p. 7).^26^ The main purpose for which empirical or, respectively, logicomathematical theories are proposed is to provide correct descriptions of the world using empirical laws or, respectively, to provide logicomathematical theorems. Given that these are the main purposes it is not surprising that Carnap regards the fruitfulness requirement as the most important requirement when explicating empirical concepts or, respectively, logicomathematical concepts. Adequate explications contribute to establishing exact scientific theories by providing fruitful concepts. How to specify the fruitfulness requirement concerning the concept of rationality will play a major role in Sect. 3.2.

#### Simplicity requirement

The simplicity requirement is meant to ensure that the simpler explicatum is preferred in cases where two competing explicata meet the first three requirements equally well. The preferred explicatum may be deemed simpler because its rules of use are simple or because “the laws connecting it with other concepts” are simple (Carnap 1950/1962, p. 7). In what follows, I neglect the simplicity requirement. This does not mean that I think that the simplicity requirement has no value; simplicity certainly has value—for instance, simpler concepts are easier to remember, and theories that make use of such concepts are usually better understood. However, we have our hands sufficiently full with the other requirements which both Carnap and I consider to be more important (see Carnap 1950/1962, p. 7).

### Short comparison

In what follows, I briefly compare (traditional) conceptual analysis and explication.

Conceptual analyses and explications are complementary methods that share the first step but proceed in different directions. If the informal clarification is carried out properly, and reveals that the concept to be clarified is exact (enough), a conceptual analysis can then do the job: Carnap (1963, pp. 933–937) grants this to conceptual analysis. If one fails to carry out this first step adequately, one might be too hasty in then trying to explicate the concept: yet it seems that where the first step is not adequately carried out, this would not count as a problem for the method of explication *in general*; rather, it would be a problem for the *specific* explication that is in question. Explications and conceptual analyses are complementary.^27^

The method of conceptual analysis does not lead to more exact concepts, because both the analysandum and the analysans are required to be synonymous. By contrast, the method of explication leads to more exact concepts by replacing the inexact (or at least insufficiently exact) explicandum by a more exact explicatum.

Conceptual analyses aim for definitions, which are supposed to be analytically true and to reflect how the terms in question are used in ordinary language. Thus, conceptual analyses require that the definition reflects the synonymy between the analysandum (i.e., the definiendum) and the analysans (i.e., the definiens).^28^ A moment’s reflection reveals that the method of conceptual analysis does not leave room for different and non-synonymous characterizations of an analysandum.

In contrast, explications may—but need not—end with definitions as well. Such definitions would be at most analytically true by *stipulation*. The explicandum and explicatum are required to be differently exact; synonymy is thus excluded. The method of explication leaves room for different non-synonymous characterizations of an explicandum, since there might be different non-synonymous adequate explicata that are (minimally) similar in use to the explicandum in question and useful for different purposes.^29^ In addition, it is crucial to keep in mind that the adequacy of a particular explication depends on the intended purposes of the explication. This leaves room for revisionary theories of a given explicandum, which might lead to different theories than those epistemologists have proposed so far.^30^

There is much more to be said about conceptual analyses and explications, and the relations and differences between them. In recent years, there has been a growing interest in methods of conceptual clarification. As a consequence, several excellent papers have been published that discuss conceptual analyses and explications in a general manner. Since my main aim is to outline the method of explication as it is applied to the concept of epistemic rationality, let’s proceed to do that.^31^

## Explicating the concept of rationality

There is an ongoing debate whether the method of explication is an appropriate method for philosophy in general.^32^ Instead of reviewing arguments for or against the method or discussing it in general I assume—for the sake of this paper—that it is in essence an adequate philosophical method. There are indeed good reasons to believe that in philosophy it has been used successfully in the past: consider, for example, Carnap’s distinction between various notions of probability^33^ and Fitelson and Hawthorne’s solution to the ravens paradox^34^. Frege’s (1884) explication of number and Tarski’s (1944) explication of truth are further examples. I myself think that the method of explication is an appropriate method for philosophy. However, it has not yet been adequately specified concerning the concept of epistemic rationality.

For our outline, let us start with the first step of an explication (i.e., the informal clarification), the identification of the targeted explicandum. I am here concerned with epistemic rationality as opposed to practical and all-things-considered rationality, which is also influenced by practical factors and, perhaps, also ethical and moral ones. As understood here, epistemic rationality applies to doxastic states as opposed to actions. I focus on rationality concerning single (categorical) beliefs instead of focusing on the rationality of entire belief systems. Future research might look at rationality concerning sets of beliefs and degree-of-belief functions. Epistemic rationality and epistemic justification are widely understood as the same thing.^35^ At this informal stage of the investigation, I do not distinguish between them, and so assume the associated concepts are identical. (On the abstract and general level of this investigation, any possible difference would not be relevant. The pluralistic picture that I draw at the end of this paper might reconcile those who see a possible difference between them.) I also do not distinguish between propositional and doxastic rationality, or justification. Such a fine-grained distinction should be drawn at a later stage when one provides specific explications of the concept of epistemic rationality. Let’s focus on the explication of the following: *it is epistemically rational for an agent* *s* *to believe a proposition* *p* *(at time* *t)*.

After the first step, we move forward to focus on the requirements on the explicata. As noted above, whether the requirements are met depends on the specific purposes that are pursued by specific explications. Nevertheless, there are certain minimal conditions that any explicatum of the concept of rationality should meet. These conditions amount to necessary conditions for rationality, so to speak. In the following, I introduce the minimal conditions that must be met to fulfill the similarity and fruitfulness requirements. In the first instance, there is no minimal condition concerning exactness that is specific to the concept of rationality: in general, we hold simply that the explicatum should be more exact than the explicandum. Furthermore, the simplicity requirement is the least important of Carnap’s requirements, as noted before. I therefore set both requirements (i.e., the exactness and the simplicity requirement) aside.

### Similarity requirements

I focus on three minimal conditions of similarity that are in some way or another accepted by most—if not all—epistemologists: the *truth-indicativity*, the *exclusivity*, and the *normativity* conditions of rationality. Further minimal conditions and more precise specifications of the conditions might also be considered: as mentioned before, an explication can be improved over time, or even replaced by others that better serve the intended purpose.

#### The truth-indicativity condition

According to the *truth-indicativity condition*, if it is rational for an agent to believe a proposition, then there is a relevant indicator in the agent’s epistemic background such that the proposition is (highly) probable given the indicator.^36^ Note that the condition is not specific about how to interpret the probabilities—objective or subjective—or what is included in the epistemic background—whether only internal or also external factors. Similarly, Steup talks about believing *p* “on a basis that properly probabilifies *S*’s belief that *p*” (Steup 2018). This condition is compatible with internalist and externalist, and evidentialist as well as reliabilist characterizations of rationality, or justification. Most, if not all, of them satisfy the truth-indicativity requirement. For evidentialists, the proposition in question would be probable given the agent’s evidence, and for reliabilists it would be probable given the agent’s belief-forming process that leads to the agent’s belief in the proposition.

Depending on the specific purpose that is pursued in presenting a theory of rationality, the truth-indicativity condition might be specified in different ways. However, since I do not aim at presenting a specific characterization of rationality, there is no need to discuss these ways in more detail.

#### The exclusivity condition

According to the *exclusivity condition*, the following holds: if it is rational for an agent to believe a proposition, then it is not rational for the agent to believe the proposition’s negation (see, on these lines, White 2013). Note three things here: first, this condition does not entail that it cannot be rational for two agents who share the same body of evidence to disagree concerning a proposition, where one agent believes the proposition and the other agent believes the proposition’s negation. The exclusivity condition does not say anything about different agents. Some epistemologists might want to strengthen this condition to exclude that it is rational for two agents to disagree when they share the same evidence. For the purpose of this paper I do not need to take sides here. Second, the exclusivity condition is compatible with a position according to which it can be *pro tanto* rational, or justified, for an agent to believe a proposition while it is also *pro tanto* rational for the agent to believe the proposition’s negation.^37^ The exclusivity condition is neutral with respect to such a position, and I am not concerned with *pro tanto rationality* here. Third, the exclusivity requirement even allows for the case that it is rational for an agent to believe a proposition while it is also rational for the agent to separately believe propositions that are jointly inconsistent with that proposition.

The exclusivity condition is especially plausible from an evidentialist point of view. Whatever account of evidential support one prefers, there is no adequate account of evidential support that allows that the agent’s (total) evidence supports both an agent’s belief in a proposition and the agent’s belief in its negation.

Things may look different from a reliabilist position. The fact that a belief in a proposition was or could be formed by a reliable process does not exclude, without further specification, that another belief in the proposition’s negation was or could be formed by another reliable process as well. However, reliabilists often defend versions of reliabilism that are in accordance with the exclusivity condition.

#### The normativity condition

Epistemic rationality, or justification, is commonly considered to be normative.^38^ The *normativity condition* says: if it is rational for an agent to believe a proposition, then to believe the proposition is *prima facie* in some way or another good, obligatory, or permitted (cf. Alston 1993, p. 530). Note the following points: first, theories of rationality that assume only that there are merely positive permissibility norms in epistemology and no positive obligations, or theories that assume that there are merely evaluations, can satisfy the normativity condition. One of these three things is *prima facie* required: goodness, obligation, or permission. Second, theories of rationality that claim that there is no specific epistemic normativity, only practical, ethical, or all-things-considered normativity, are compatible with this condition as well. The condition does not restrict the normativity to epistemic normativity. Third, the condition does not exclude that there are situations where it is neither good, obligatory, nor permitted to believe a proposition, even though it is rational for the agent to believe it. According to the normativity condition, to believe a proposition which it is rational to believe is only *prima facie* good, obligatory, or permitted. It is not required that it is in every situation good, obligatory, or permitted.

Any explication of the concept of rationality has to consider the *truth-indicativity*, the *exclusivity*, and the *normativity* conditions of rationality. For the purposes of this paper, I assume that if one does not accept these three minimal conditions, then one is targeting another concept.

### Fruitfulness requirements

Carnap states the fruitfulness requirement for explication as follows:

The explicatum is to be a *fruitful* concept, that is, useful for the formulation of many universal statements (empirical laws in the case of a nonlogical concept, logical theorems in the case of a logical concept). (Carnap 1950/1962, p. 7)

Carnap focuses on inexact *empirical* or inexact *logicomathematical* concepts. I focus on normative concepts. (Shepherd and Justus go so far as to complain that “Carnap said nothing about how fruitfulness should be conceptualized for [normative] concepts” (2015, p. 282).) Concerning empirical and logicomathematical concepts, the attractiveness of Carnap’s account of fruitfulness is based on the purpose for which the *empirical* or, respectively, *logicomathematical theories* are proposed, namely to provide correct descriptions of the world using empirical laws, or respectively to generate logicomathematical theorems.

How can we determine what the epistemic counterparts of empirical laws or logical theorems are in the case of explications of the concept of (epistemic) rationality? With Shepherd and Justus (2015), I propose that we should determine the counterparts by concentrating on the intended purposes, or goals, of theories of rationality and of the associated explications. This is in line with both Carnap (1963) and Quine (1960/2013), who emphazise that the purpose for which an explication is provided is to be taken into account when explicating the concept. What are these purposes? Unfortunately, Shepherd and Justus (2015) do not have anything specific to offer for the concept of epistemic rationality. They focus on epistemic concepts in general, but not on the concept of epistemic rationality in particular. However, in connection with the fruitfulness requirement, they briefly mention the purpose of guidance. Following and extending this idea, I focus on two popular (kinds of) purposes, although in doing so, I do not want to suggest that other purposes found in the literature are not relevant—however, this is not the place to discuss all the possible kinds of intended purposes. I concentrate on the following ones: first, the purpose of *guiding* the formation (or maintenance) of doxastic states in an adequate way; second, the purpose of *assessing* (the formation or maintenance of) doxastic states in an adequate way.^39^ An adequate theory of rationality assesses doxastic states or guides their formation by implying normative statements that adequately assess doxastic states or appropriately guide their formation. Thus, I propose that the counterparts of empirical laws or logical theorems are general norms and evaluations, which guide and assess doxastic states. For the explicatum of the concept of rationality to be sufficiently fruitful, it should be “useful for the formulation of many [acceptable] universal” *epistemically normative statements*, epistemic evaluations or epistemic norms. In some way or another, theories of rationality are proposed to systematize such evaluations and norms. This fits well with the normativity condition that I introduced before, as well as with our instrumentalism concerning the clarification of the concepts of rationality. In their discussion of the method of explication in epistemology, Shepherd and Justus (2015) also adopt an instrumentalist position concerning conceptual clarifications. As a consequence, Shepherd and Justus also favor instrumentalist positions about epistemic rationality, such as Brössel’s, mine, and Huber’s (2013). In particular, they think that the fruitfulness condition should take into account that epistemic theories concern the achievement of epistemic ends (Shepherd and Justus 2015: Sect. 5.1). Thus, Shepherd and Justus’s approach indicates how an instrumentalist position about theories of rationality leaves room for an instrumentalist position about epistemic rationality itself. Here I do not focus on instrumentalist positions about epistemic rationality any further; I discuss such positions in Brössel et al. (2013).

## Purposes of theories of rationality: a pluralistic picture

My main aim is to outline the method of explication as it should be applied to the concept of epistemic rationality. Whether a theory of rationality and the explication in question is adequate depends on the intended purposes connected with them. As I see it, these purposes are relevant for whether the explicanda satisfy the similarity, exactness, fruitfulness, and simplicity requirements, which fits with Carnap (1963) and Quine (1960/2013). I propose placing these purposes front and center. Thus, in what follows I focus on the purposes in more detail.

### Purpose of guiding

According to *guidance accounts* the following holds:

***Guidance***: A (primary) purpose of theories of rationality is that of *guiding* the formation (or maintenance) of doxastic states.

Theories of rationality that are instrumental for guiding the formation of doxastic states do so by saying what *epistemic norms* follow from the claim that it is rational for an agent to believe a proposition. (They do so, for example with the help of bridge principles, in order to satisfy the *normativity condition* (see Sect. 3.1). This condition requires that if it is rational for an agent to believe a proposition, then to believe the proposition is *prima facie* in some way or another good, obligatory, or permitted. In the spirit of the purpose of guiding the formation of doxastic states, such bridge principles might establish a normative connection between rational doxastic states and norms.) Such norms are expressed in terms of deontic phrases such as: ‘ought to’ or ‘ought to see to it that’, ‘is permitted’, or ‘is forbidden’.^40^ It is such norms that, strictly speaking, guide the formation of doxastic states. Accordingly, an explication of the concept of rationality that is proposed as providing guidance is fruitful when it is useful for the formulation of general epistemic norms. The norms, then, are the counterparts of empirical laws or logical theorems.

In what follows I present different conceptions of guidance. Note that I will not clarify the concept of guidance by going through each step of an explication; I hope that my account of it will be sufficiently clear. Furthermore, explicating the concept of guidance would presuppose more basic purposes (than the purpose relevant for devising theories of rationality) with respect to which the concept of guidance would have to be fruitful. In this paper, I do not want to speculate what these more basic purposes might be. The same holds for the concept of assessment that I discuss later.

Now let us consider different conceptions of guidance. I will review objections to them, defend a specific conception for the present purpose, and argue that the mentioned objections do not threaten it. It is helpful to discuss the different conceptions with respect to how they deal with the following requirement:^41^

***Can***: An epistemic ought-norm of the form ‘*s* ought to believe that *p* (given circumstance or input condition *c*)’ guides the formation of an agent *s*’s doxastic state in an adequate way only *if* *s* *can (in principle) form the doxastic state*.^42^

The conception of guidance that is of interest here satisfies this minimal condition. Now, though, we must ask how ‘can’ is to be understood in *Can*.

#### The non-violation conception of guidance

According to the *non-violation conception* of guidance, the if-clause in *Can* is read as: ‘if it is logically possible that *s* forms the doxastic state’ or ‘if it is metaphysically possible that *s* forms the doxastic state’.^43^ According to this conception, one can be guided by a norm by mere luck, by not violating it, and by luckily forming a belief that conforms with the norm. This conception of guidance is implausible and leads to the mentioned *problem of luck*. I am not looking for such a conception. It takes more to be guided by a norm than simply not violating it. Rather, I am looking for a more demanding conception of guidance, according to which *Can* requires that an epistemic norm is considered to guide the formation of an agent’s doxastic states only if she can (in principle) form the doxastic state *by following the norm*: that is to say, by applying it as a rule.

#### The intellectualist conception of guidance

The best-known conception of guidance is the *intellectualist conception*.^44^ According to this conception, the if-clause in *Can* is read as: ‘if *s* can deliberately form the doxastic state (i.e., by exercising direct and voluntary control)’. As per this conception, the formation of one’s doxastic state is guided by norms in such a way that one directly and voluntarily controls the formation of each doxastic state by thinking about the norm and deliberately following it. “This model assimilates the functioning of epistemic norms to the functioning of explicitly articulated norms” (Pollock 1987, p. 64).

Some opponents of guidance accounts seem to have this conception in mind when they argue against such accounts along the following lines:

**Table:** 

(1)	Guidance accounts are correct.
(2)	If guidance accounts are correct, then a purpose of a theory of rationality is that of guiding the formation of doxastic states.
(3)	If a purpose of a theory of rationality is that of guiding the formation of doxastic states, then there are epistemic norms that guide the formation of doxastic states.
(4)	There are epistemic norms that guide the formation of doxastic states only if agents can *directly voluntarily control* the formation of their doxastic states by following the norms in question.
(5)	However, agents cannot directly voluntarily control the formation of their doxastic states.
($\therefore$)	Guidance accounts are not correct.

Such arguments (and variations thereof) are in the spirit of Alston (1986) and Goldman (1980), and exemplify the *voluntary-control problem*. In brief, the problem is that guidance accounts seem pointless: since agents cannot have direct voluntary control over the formation of their doxastic states, they cannot therefore be guided by the associated norms. Yet even if successful in arguing against the intellectualist conception, the problem does not force us to reject guidance accounts altogether, for there are yet other conceptions of guidance that do not require that agents can have direct voluntary control over the formation of their doxastic states.

#### The loosely regulative conception of guidance

According to what I call the *loosely regulative conception* of guidance, which can be found in Spohn (2012: Sect. 11.6) and is (implicitly) assumed by others, the if-clause in *Can* is read as: ‘if *s* can attempt to form the doxastic state’ or the slightly stronger ‘if *s* can attempt to do one’s, *s*’s, best to form the doxastic state’.^45^ According to this conception, norms are meant to regulate the formation of one’s doxastic state in some vague manner. *Can* is not to be taken strictly (Spohn 2012, pp. 259–260). Spohn says in this respect: “We might at first have no idea how to realize the norms we accept. We may realize them only roughly. Or we may only attempt to realize them and do something that at least moves us in the right direction” (Spohn 2012, p. 259).

The loosely regulative conception suffers from what I refer to as the *trivialization problem*: in some way or another, it trivializes *Can*. It strikes me as trivially true that we may “attempt to [...] do something that at least moves us in the right direction”. Without further specification of what ‘attempt to do’ or ‘attempt to do one’s best’ means, it seems that even coma patients might attempt to do their best to believe all logically true propositions. As in the case of the non-violation conception, according to the loosely regulative conception it is not excluded that there are adequate epistemic norms that hardly offer any guidance; but according to the loosely regulative conception they do provide guidance. For instance, according to the loosely regulative conception, norms of the following form guide the formation of our doxastic states: ‘*s* ought to believe all and only true propositions’. However, norms such as ‘*s* ought to believe all and only true propositions’ do not offer any assistance in the formation of doxastic states such that one fully satisfies it; indeed no rule can guide one to satisfy the norm ‘*s* ought to believe all and only true propositions’. Admittedly, some norms or rules might help to come close(r) to believing all and only true propositions; they might offer some loose orientation or regulation. Still, they offer hardly any assistance in fully satisfying the norm since it is not humanly attainable to believe all and only true propositions. Worse, one might not, even in principle, have direct access to all factors that are relevant for conforming to this norm—e.g., the input conditions of norms—and moreover one simply does not have the cognitive capacity to conform to it even remotely (to believe all and only true propositions). Spohn accepts norms such as ‘be consistent!’ and ‘believe only truths and as many of them as possible!’,^46^ because in some way or another they offer orientation or regulation (Spohn 2012, pp. 260–261). He says that the norm ‘believe only truths and as many of them as possible!’ “is a perfectly good norm even if we had no methodology for searching for truths and no criterion for distinguishing between truths and falsehoods” (2012, p. 260). Here I disagree: to be guided by a norm, and thus to be able to follow it, it is required that there is at least a rule or a “methodology”—the norm itself or another one—that assists one in satisfying the norm. It is perfectly fine if there are other different such rules or methodologies, each of which assists one in satisfying the norm. What I consider important is that there is at least one such rule or methodology.

With Spohn, one might have in mind a notion of guidance that is hardly demanding. However, if there is no rule that provides guidance in a more demanding sense, then one had better formulate the norm in question as an evaluation, which assesses and, in virtue of so doing, in some way or another gives orientation and guides in a loosely regulative way. Accordingly, ‘believe only truths and as many of them as possible!’ could be intended to be synonymous with epistemic evaluations such as ‘it would be good if you believed all and only true propositions’, ‘it would be ideal if you believed all and only true propositions’, or ‘it would be best if you believed all and only true propositions’. Spohn might agree to rephrase the respective norm in evaluative terms: although he does not consider the different functions of epistemic evaluations and epistemic norms, he distinguishes what I call *levels of normativity*, which—charitably interpreted—correspond more or less to the assessment and guidance function of evaluations and norms. Spohn says:

[...] I have refrained from a positive specification of the *regulative level*. Anything that may be plausibly advanced in a normative discussion is fine; and if it is not a directly accessible and applicable rule or procedure, it belongs to the *regulative level*. (Spohn 2012, p. 261; my emphasis)

Instead of distinguishing between levels of normativity, I propose to distinguish epistemic evaluations and epistemic norms, analogously to what we do in ethics. Epistemic evaluations assess doxastic states and roughly correspond to the “regulative level”. In a very loose sense, evaluations also give orientation and regulate by setting standards. Epistemic norms guide the formation of doxastic states, and they roughly correspond to the level of “directly accessible and applicable rule[s]”.

My distinction between evaluations and norms helps to prevent confusion, and also to avoid certain conflicts of norms. Presumably, for instance, according to the loosely regulative conception, the following norms are both correct: ‘an agent *s* ought to believe all and only true propositions’ and ‘an agent *s* ought to believe a proposition just in case the proposition (or belief in it) is supported by the agent’s total evidence.’ However, in many cases, one may form one’s belief by following the second norm and by doing so violate the first norm—just consider cases where a false proposition (or belief in it) is supported by one’s total evidence. I refer to this problem as the *problem of conflicts of norms*. However, with my approach the conflict can be resolved in a natural way. Let’s take the distinction between epistemic evaluations and epistemic norms for granted; based on this distinction, then, one might endorse the latter norm, ‘an agent *s* ought to believe a proposition just in case the proposition is supported by the agent’s total evidence’, and rephrase the former as the following epistemic evaluation: ‘an agent *s* ideally believes all and only true propositions’. No conflict of norms arises!^47^

#### The internalization conception of guidance

Pollock’s (1987) *internalization conception* is an attractive alternative to the conceptions that I have discussed so far.^48^ The purpose of guiding has enjoyed much attention in general, but most epistemologists have ignored the internalization conception of guidance.^49^ According to it, the if-clause in *Can* is read as: ‘if *s* can form the doxastic state by having internalized the guiding norm’.

According to Pollock’s conception, if an epistemic norm guides the formation of an agent’s doxastic state, then the agent can follow the epistemic norm by having internalized it. As a consequence, one can form a doxastic state by following norms although one may not do it deliberatively—one might not “think about them” (Pollock 1987, p. 68). In this respect, it is like following linguistic rules. We follow linguistic rules all the time without deliberatively following them, sometimes even without being able to state them explicitly.^50^

One can be guided by an (epistemic) norm by having internalized the norm by knowing *how* to follow it, although one may not be in a position to state the norm or always to deliberatively form the correct doxastic state. I agree with Pollock, who says:

To say that we know how to reason is to invoke a competence/performance distinction. It in no way precludes our making mistakes. It does not even preclude our almost always making mistakes in specific kinds of reasoning. All it requires is that we can, in principle, discover the errors of our ways and correct them. (Pollock 1987: En. 8)^51^

I take it for granted that human agents have the cognitive abilities and faculties to internalize the norms in question, that they are in principle cognitively able to follow them, and have direct access to all factors—e.g., input conditions of the norms—that are relevant. (The internalization process may take a while—just as the internalization of linguistic rules takes a while.) According to Pollock, “[t]he sense in which they must be directly accessible is that our automatic processing system must be able to access them without our first having to make a *judgment* about whether we are in circumstances of [the required] type. We must have non-epistemic access” (Pollock 1987, p. 69). I think that if agents do not have the cognitive abilities and faculties, nor the relevant access required to follow a specific norm, then the norm is just not adequate. (According to how I understand the internalization conception of guidance, a norm also counts as internalized if one internalizes a second norm which assists one in satisfying the first norm. Here my position might depart from Pollock’s.)

It should be clear by now that—in contrast to the intellectualist conception—the internalization conception does not require that one has direct voluntary control over the formation of one’s doxastic states. Since it is not the case that epistemic norms guide the formation of doxastic states only if agents can *directly voluntarily control* the formation of their doxastic states, the voluntary control problem does not arise.

I adopt Pollock’s internalization conception of guidance. The question then arises: To what extent does this choice of conception have any bearing on which epistemic norms are correct? The relevance of the choice can be seen when one adopts different perspectives: a *first-* or *third-person perspective*.^52^ One adopts a *first-person perspective* just in case one adopts a perspective from which one exclusively takes into account factors to which the agent has direct access (see Pollock 1987: Sect. 3)). I do not take any stance here concerning the question of whether such factors have to be internal factors or can also be external. Traditionally, they are considered to be internal.^53^ However, for the points I want to make it is not crucial whether they are conceived as internal. By contrast, one adopts a *third-person perspective* just in case one adopts a perspective from which one also takes factors into account to which the agent has no direct access. For instance, they can be facts about whether a given doxastic state towards a proposition was reliably formed.

A norm that appeals to factors to which one does not have direct access when forming one’s doxastic state cannot be followed. Acceptable epistemic norms, then, appeal exclusively to factors to which we have direct access. The formation of one’s doxastic state can be guided from a *first-person perspective* only (see also Pollock 1987). This restricts our choice of explicanda and, thus, our choice of characterizations of rationality, or justification. It seems only natural to say that if a theory of rationality is meant to guide the formation of doxastic states, this is to be done from the first-person perspective, that is by considering only directly accessible factors.^54^

If a theory of rationality is meant to guide the formation of doxastic states, which is done from the first-person perspective, then the theory guides the formation of doxastic states by implying norms that provide such guidance. And since from such a perspective only directly accessible factors are to be considered, the particular theory cannot be reliabilist. Thus, the choice of the purpose for which a theory of rationality is proposed has a bearing on explications of the concept of rationality and theories of rationality.

### Purpose of assessing

How do things look when one considers the purpose of assessing doxastic states? In the current section we focus on accounts that deal with this purpose. As it will turn out, assessment accounts are less restrictive.

The following is characteristic for *assessment accounts*:

***Assessment***: A (primary) purpose of theories of rationality is that of *assessing* (the formation or maintenance of) doxastic states.

Theories of rationality that are instrumental for assessing doxastic states do so by saying what *epistemic evaluations* follow from the claim that it is rational for an agent to believe a proposition. (Similar to before, they do so, for example with the help of bridge principles, in order to satisfy the *normativity condition*. In the spirit of the purpose of assessing doxastic states, such bridge principles might establish an evaluative connection between rational doxastic states and evaluations.) Such evaluations are expressed in terms of axiological phrases such as ‘it is good to’, ‘it is valuable that’, or ‘it is bad to’. It is the evaluations that, strictly speaking, assess doxastic states. Accordingly, an explication of the concept of rationality that is proposed to assess such states is fruitful when it is useful for the formulation of general epistemic evaluations. Such evaluations are, then, the counterparts of empirical laws or logical theorems in the case of explications of empirical or logicomathematical concepts.

For adequate conceptions of assessment, no condition is analogous to *Can*. According to the assessment conception I envisage here, an agent’s capacities or limitations are not taken into account. In particular, it is not required that the agent can form the doxastic state that is evaluated as being good, ideal, or best. This conception allows me to contrast assessment accounts with guidance accounts in a transparent way. However, there is also a relativized notion of assessment to which I am sympathetic. According to it, one assesses an agent’s doxastic states relative to the agent’s capacities or limitations. To use a slight modification of an example by David Christensen, one might assess a student’s often erroneous beliefs concerning mathematical statements as good relative to the student’s cognitive abilities and faculties, although one would not do so in the same way if they were the beliefs of an expert mathematician or logician. There is a “fundamental metric” or “absolute scale”—to borrow Christensen’s terms—that allows us to compare both doxastic states in such a way that the mathematician’s beliefs concerning mathematical statements are better than the student’s (Christensen 2004, p. 163). Here I presuppose such a fundamental metric.

An agent’s doxastic states can be assessed from different perspectives: first- or third-person. The first-person perspective is certainly relevant for reflections about one’s past and present doxastic states, which is crucial for whether one should stick to them and for whether one should collect new evidence. Along these lines, Laurence BonJour says: “Part of one’s epistemic duty is to reflect critically upon one’s beliefs, and such critical reflection precludes believing things to which one has, to one’s knowledge, no reliable means of epistemic access” (BonJour 1985, p. 42). If the purpose for which a theory of rationality is proposed is that of assessing doxastic states from a first-person perspective, only those factors are to be considered to which the agent in question has (direct) access.

The assessment provided by a theory of rationality can also adopt a third-person perspective. As mentioned above, from a *third-person perspective* one also takes factors into account to which the agent in question does not have (direct) access.

According to Pollock, assessing doxastic states, especially from a third-person perspective, plays no prominent role in epistemology. Pollock only considers the purpose of guiding the formation of doxastic states—as opposed to the purpose of assessing them—to be relevant in epistemology. However, he admits that assessment from a third-person perspective is sometimes helpful for practical purposes: to borrow his example, assessing scientists’ doxastic performance (concerning a specific research area)^55^ from a third-person perspective is relevant in situations wherein one needs to hire a scientist (Pollock 1987, p. 64). Yet Pollock errs in saying that assessments from a third-person perspective play no prominent role in epistemology. There is no doubt that although the final need or desire in the case of the scientist’s hiring might be a practical one and that practical considerations should also be taken into account, the scientist’s scientific performance should nevertheless be assessed from an epistemic point of view.

Furthermore, opponents who claim—as does Pollock—that the third-person perspective is not relevant in epistemology overlook the relevance of this perspective in social epistemology. Social epistemology focuses on doxastic states of communities or of single agents *qua* members of communities. It deals with questions such as: With whom should one collaborate in one’s epistemic endeavors? What makes one an epistemic expert or peer (concerning a subject area)? When is one permitted to rely on the beliefs or testimonies of experts? In answering such questions, it is relevant to assess other agents’ doxastic states from a third-person perspective. The assessment of other agents’ doxastic states from a third-person perspective is thus sometimes relevant for forming one’s doxastic states. For instance, scientists specialize, divide their epistemic labor, and so pursue their epistemic endeavors more efficiently. In doing so, they often rely on the testimony of fellow scientists. Whether one should consider another agent’s doxastic states when forming one’s own doxastic states—and, if so, how—may depend on how one assesses her former doxastic states. For instance, whether one should trust a colleague’s testimony, or whether one should change one’s doxastic state towards a proposition after disagreeing with someone about it, may depend on how one assesses the agent’s former doxastic states from one’s own perspective, which is a third-person perspective. One should thereby consider all one’s available information, which also includes factors that are not accessible to the agent whose doxastic state is assessed. Such information might be information on whether the other agent’s doxastic states on the subject area were formed by reliable processes (related to this see Greco 2008, pp. 266–267).

Thus it seems only natural to say that if a theory of rationality is meant to be instrumental for assessing doxastic states from the first-person perspective, then the theory assesses doxastic states by implying evaluations that assess doxastic states from a first-person perspective. Such a theory cannot be reliabilist, because the theory can consider only directly accessible factors. By contrast, if a theory of rationality is meant to be instrumental for assessing doxastic states from the third-person perspective, then the theory assesses doxastic states by implying evaluations that adequately assess doxastic states from a third-person perspective, and both evidentialist as well as reliabilist characterizations are possible, as well as certain other kinds of theories that also consider factors that are not directly accessible. As mentioned before, those characterizations and associated theories can be revisionary in nature.

### A pluralistic picture

The general picture that I want to advocate is pluralistic. The method of explication does not exclude that there are different adequate explications of the (original) concept of rationality. Different purposes might call for different explications and characterizations, which can exist side by side, since they are designed for different purposes. My position allows for a division of labor between theories of rationality: when the purpose for which a theory of rationality is proposed is that of guiding the formation of doxastic states, the characterization of rationality is not reliabilist: only directly accessible factors can be considered. However, if the purpose is that of assessing doxastic states, the characterization of rationality can be evidentialist or reliabilist, or indeed draw on some other kind of theory altogether. The theory might consider only directly accessible factors as well as factors which are not directly accessible.

Depending on the specific intended purposes, one might end up defending revisionary theories, which might be evidentialist or reliabilist, or neither. That one might end up defending revisionary theories is so because the focus is on the purposes, something which has been overlooked by most epistemologists who are concerned with theories of rationality. Such philosophers have usually begun by presenting an analysis of the concept of rationality, and only after that looked into the purposes that the theory fulfills. In contrast, I have proposed placing those purposes front and center.

## Conclusion

I have proposed that the concept of rationality be explicated, thereby exploring a method of conceptual clarification that many epistemologists have neglected. For an (adequate) explication of the concept of rationality, the decisive considerations turn out to be the normativity of rationality as well as the purpose for which the particular theory of rationality is proposed. Since purposes, or goals, play such a determinate role in the explication of the concept of rationality and the characterization of rationality, my position amounts to an instrumentalist position about theories of rationality. Given the wide range of purposes which could be appealed to, the method of explication leaves room for different characterizations of rationality, and thus for epistemic pluralism. However, in order to be adequate as an explication of the concept of rationality, the explicatum is still required to satisfy the conditions of truth-indicativity, exclusivity, and normativity, and should be “useful for the formulation of many [acceptable] universal” norms or evaluations.

## Footnotes

1 Since I here focus exclusively on epistemic rationality, I often omit the reference to the epistemic dimension.

2 For a discussion of the philosophy and historical development of conceptual analysis, see Beaney (2018).

3 I remain as neutral as possible on what concepts are. I think that what I say is useful for different approaches towards clarifications of the concept of rationality. This is so independently of which understanding of concepts is in play. However, it seems reasonably safe to understand concepts as, roughly, the meanings of predicates.

4 For an account of how one might proceed step-by-step in providing an explication, which is not restricted to explications of the concept of epistemic rationality, see Cordes (2020), and Cordes and Siegwart (2018: Sect. 2). Such procedural accounts of explication trace back to Hanna (1968). Here we have our hands sufficiently full with providing an outline of the method of explication as it applies to the concept of rationality. A detailed, step-by-step description of the process of explication might be helpful at a later stage when we come to focus on specific explication(s) of the concept of rationality.

5 Susanne Hahn also bases her investigation on the method of explication, but focuses on the concept of practical rationality. She describes her approach as delivering a “country map” rather than a “hiking map” for explications of the concept of rationality (Hahn 2013, p. 20). Here I seek to do the same for the concept of epistemic rationality. (Hahn focuses on practical rationality, but briefly deals with epistemic rationality as well, although she does not provide an explication of the latter concept. For her purposes, and given the structure of her book, it suffices to speak of the rules that permit certain kinds of speech acts associated with the various kinds of graded propositional attitudes. The set of rules is to be evaluated as adequate (or not) with regards to whether it furthers the ends in question (Hahn 2013: Sect. 14.3)).

6 Shepherd and Justus (2015) also emphasize that in epistemology, the method of explication has to do justice to the normativity of epistemic concepts. In Sect. 3.2, I say more about their account and the way it differs from mine.

7 This instrumentalist position about theories of rationality leaves room for instrumentalist positions about rationality itself (see Sect. 3.2 here).

8 Beaney (2018) and Olsson (2017: Sect. 3) provide an overview of such alternative methods.

9 Alston (1993) makes similar points. He mentions the disagreement and thinks that there is more than one concept of justification. Based on this he establishes his version of epistemic pluralism. For a short but instructive comparison between Alston’s epistemic pluralism and the method of explication, see Olsson (2017), who also discusses the method of explication and epistemic pluralism in general.

10 For more details on inexactness in the context of explication, see (Hahn 2013: Sect. 2.3).

11 Similar to me, Hahn also thinks that the concept of rationality is “semantically defective” (2013, p. 383).

12 As a reviewer of this paper rightly pointed out, there are concepts in the vicinity of the concept of epistemic rationality (as applied to belief or believing), such as the concept of plausibility or of credibility, for which this second shortcoming might not apply.

13 There are a few philosophers who have focused on this method in epistemology [e.g., Hahn (2013: Sect. 14.3), Kitsik (2020), Lehrer (1990), Maher (2007), Olsson (2015 and 2017), Shepherd and Justus (2015)]. In contrast to the approach here, almost all such approaches concern the concept of knowledge, or address debates in formal epistemology. To my knowledge, besides Hahn (cf., Fn. 5 in this paper), Olsson (2017) is the only one who explicitly addresses explications of the concept of (epistemic) rationality, or justification; yet he focuses only on explications in epistemology in general. There is no room here to compare all these approaches with mine.

14 For a thorough presentation of the pre-history of the method of explication as it is understood here, see Cordes and Siegwart (2018: Sect. 1).

15 See Carnap (1950/1962, pp. 3, 5, 7, 1963, p. 935; and 1988, pp. 7–8), Quine (1960/2013, p. 241), Hempel (1950, p. 154), and, similarly, Hempel (1952, p. 11). It is noteworthy that Carnap first says that an exact concept should replace the explicandum (Carnap 1950/1962, p. 3); later he claims only that it has to be replaced by a *more* exact concept (Carnap 1963, pp. 935–936). Furthermore, I think that exactness is to be understood as exactness relative to specific purposes; this is in line with Carnap (1963).

16 For more on this, see Brun (2016, 2020).

17 For an instructive presentation of conceptual ethics and its significance in philosophy, see Burgess and Plunkett (2013a, 2013b).

18 Note that in this paper, ‘conceptual clarification’ refers to entire clarifications while ‘informal clarification’ refers to the first steps of explications and conceptual analyses.

19 See Carnap (1963, p. 935), similarly see Maher (2007, p. 336).

20 This is also noted by Brun (2016, p. 1230), Justus (2012, p. 165) and Maher (2007, pp. 335–336). However, some philosophers also falsely assume that providing a definition is necessarily a part of an explication (e.g., Boniolo 2003).

21 One of these being Boniolo (2003, pp. 291–292).

22 See, similarly, Olsson (2017).

23 Cordes and Siegwart even claim that the “the pragmatic outlook [concerning explications] with its rather liberal selection of functional criteria of adequacy is more pronounced in Quine than in Carnap” (2018: Sect. b.). This is emphasized in Quine (1960/2013) and prominently adopted in Carnap (1963). Not only Carnap and Quine but also Hempel (1952: Ch. I) emphasizes the purposes of explications.

24 I am sympathetic to Quine’s account of similarity. He understands it pragmatically, in terms of the function of explicanda (Quine 1960/2013, p. 241). For a more detailed discussion of Quine’s similarity condition, see Cordes and Siegwart (2018: Sect. b.)

25 See also Reck (2012: Part 1).

26 Along similar lines, Hempel emphasizes that whether a “proposed system of concepts” is justified depends on its “ability to serve in the formulation of sound and comprehensive generalizations” (Hempel 1950, p. 154).

27 See Carnap (1963, p. 940); similarly Strawson (1963, pp. 517–518).

28 As noted by two reviewers of this paper, the required synonymy leads to the well-known *Paradox of Analysis*. Langford states the paradox as follows: “[I]f the verbal expression representing the analysandum has the same meaning as the verbal expression representing the analysans, the analysis states a bare identity and is trivial; but if the two verbal expressions do not have the same meaning, the analysis is incorrect” (1942, p. 323). Since I do not want to defend the method of conceptual analysis, nor do I focus on it, I leave it to the aficionados of this method to defend it in the light of this paradox. The paradox does not arise for explications since they do not require synonymy between the explicandum and the explicatum. For a more detailed discussion of the paradox, see Carnap (1988), Cordes and Siegwart (2018), and Olsson (2017). To discuss the paradox any further would take us too far from my main aim.

29 Brössel (2013, p. 393), Justus (2012, p. 168), and Olsson (2017) also note this. Carnap himself proposes two explicata for the concept of probability, namely ‘$\hbox {probability}_1$’ which refers to the degree of confirmation and ‘$\hbox {probability}_2$’ which refers to relative frequency (1950/1962, pp. 23, 25–26). He also proposes two explicata for the concept of confirmation (and for its classificatory, comparative, and quantitative variations), the first being ‘firmness’ which refers to absolute confirmation, the second ‘increase in firmness’ which refers to incremental confirmation (Carnap 1962: xvi; note that this reference to firmness and increase in firmness is new in the preface of the 1962 version).

30 Dutilh Novaes (2020) and Kitsik (2020) present instructive discussions of the revisionary potential of explications.

31 For a thorough discussion of analyses in general, see Beaney (2018); and for an insightful presentation of the method of explication in general, see Cordes and Siegwart (2018). Both texts are systematically insightful as well as rich in historical remarks. See also Eder et al. (2020a) collection of recent papers on philosophical methods, some of which focus on the method of explication in general [see, for example, Brun (2020), Cordes (2020), Dutilh Novaes (2020), and Pinder (2020)]. See also the collection’s introduction, Eder et al. (2020b).

32 For arguments *against* the method of explication as applied to philosophy, see, for example, Boniolo (2003) and Strawson (1963). For accounts in *favor* or in *defense* of it, see Brun (2016, 2020), Dutilh Novaes (2020), Dutilh Novaes and Reck (2017), Maher (2007), Olsson (2015, 2017), Justus (2012), Shepherd and Justus (2015), Kitsik (2020), and Schupbach (2017). Pinder (2020) explores the limits and prospects of the method of explication in philosophy.

33 Carnap (1950/1962, p. 23 and pp. 25–26).

34 One possible diagnosis of Hempel’s ravens paradox is that it arises due to an inexact concept of confirmation—as it is used in ordinary language. However, if one explicates the concept in probabilistic terms, the paradox can be resolved (see Fitelson and Hawthorne 2010).

35 See, e.g., (Cohen 1984, p. 283); (Smithies 2012, p. 274); (Wedgwood 2012, pp. 274, 280).

36 Similarly, Alston (1993, p. 528f).

37 For an informative discussion of *pro tanto* justification, see Graham (2006).

38 For literature that takes (epistemic) rationality to be normative or even claims that this is widely assumed see, for instance, Kelly (2003), Brössel et al. (2013), Eder (2020), Wedgwood (2018).

39 Among others, Goldman (1980) and Pollock (1987) discuss such purposes of theories of rationality. Goldman sides with approaches that assume that the (main) concern of theories of justification is to assess doxastic states, while Pollock sides with approaches that assume that the (main) concern of theories of justification or the corresponding norms is to guide the formation of doxastic states. Goldman’s and Pollock’s approaches are very different from mine, as will become apparent to connoisseurs of these approaches (see Sects. 3 and 4 here).

40 There are different readings of ‘ought’ in ordinary language, *evaluative* and *deliberative* (see Schroeder (2011) and Wedgwood (2007)). In this paper, I disregard evaluative readings, and I assume that *deontic* phrases such as the ones mentioned above are used with a guidance function rather than an evaluative function. For evaluative functions I consider *axiological* phrases such as ‘it is good to’, ‘it is valuable that’, or ‘it is bad to’.

41 There might be another conception of guidance according to which *Can* does not hold. According to such a conception, guidance is very loose; it has already been provided when some regulation or orientation has been given, independently of whether one can (in principle) form the respective doxastic state. Indeed, the *loosely regulative conception* of guidance that I will shortly discuss might not be in accordance with *Can*. The reason why I am interested in a more demanding notion of guidance is that we have evaluations that provide loose guidance. If the conception of guidance that we are looking for were such a conception of loose guidance, it seems there would be hardly any significant difference between guidance and assessment.

42 I think that this condition motivates the well-known *ought-implies-can principle*, which says that if one ought to do this or that, one can do this or that; but to argue for this here would take us too far afield.

43 See Chuard and Southwood 2009: Sect. 5 for a discussion of this conception or the abovementioned readings of ‘can.’ Greco (2010: Ch. 2) discusses a similar account.

44 The label can be traced back to Pollock, who criticizes this conception (1987, p. 65).

45 Wolfgang Spohn in conversation.

46 Strictly speaking, these are not norms as understood here because they do not contain deontic phrases such as the ones mentioned at the beginning of Sect. 4.1: ‘ought to’, ‘ought to see to it that’, ‘is permitted’, or ‘is forbidden’. Assuming a charitable reading, however, they are synonymous with ‘You ought to be consistent’ and ‘You ought to believe only truths and as many of them as possible,’ respectively. Thus, I set aside that they are not norms in the stricter sense here.

47 I am not committed to the view that there are no conflicts of norms, but I think that they should be avoided whenever possible.

48 Although I adopt Pollock’s conception of guidance as described here, I remain neutral on his naturalistic internalist account, according to which epistemic norms are the norms that *actually* guide our reasoning.

49 The few exceptions include Pryor (2008, p. 196), who endorses this conception; and Spohn (2012: Sect. 11.6), who does not.

50 Pollock’s internalization conception of guidance seems to trace back to Wittgenstein’s (1986, pp. 85) account of following rules.

51 Furthermore, Pollock’s competence/performance distinction is analogous to Chomsky’s (1965, pp. 3–4) competence/performance distinction in linguistics.

52 I think that the difference between second- and third-person perspectives is not crucial in the present general context, where specific norms are not discussed. From both perspectives, one takes into account factors to which the agent in question does not have direct access. For simplicity, I exclusively focus on first- and third-person perspectives.

53 Pollock (1987) is famous for arguing that they are internal.

54 A moment’s reflection reveals that this would not necessarily be so if the non-violation or the loosely regulative conception of guidance were correct.

55 To be more precise, Pollock talks about assessing a scientist as a scientist; however, I think this can be neglected here.

## References

[CR1] Alston W (1986). Internalism and externalism in epistemology. Philosophical Topics.

[CR2] Alston W (1993). Epistemic desiderata. Philosophy and Phenomenological Research.

[CR3] Beaney, M. (2018). Analysis. In: E.N. Zalta (Ed.), *The Stanford Encyclopedia of Philosophy* (Summer 2018 Edition). https://plato.stanford.edu/archives/sum2018/entries/analysis/.

[CR4] Boniolo G (2003). Kant’s explication and Carnap’s explication: The redde rationem. International Philosophical Quarterly.

[CR5] BonJour L (1985). The structure of empirical knowledge.

[CR6] Brössel P (2013). The problem of measure sensitivity redux. Philosophy of Science.

[CR7] Brössel P, Eder AMA, Huber F (2013). Evidential support and instrumental rationality. Philosophy and Phenomenological Research.

[CR8] Brun G (2016). Explication as a method of conceptual re-engineering. Erkenntnis.

[CR9] Brun G (2020). Conceptual re-engineering: from explication to reflective equilibrium. Synthese.

[CR10] Burgess A, Plunkett D (2013). Conceptual ethics I. Philosophy Compass.

[CR11] Burgess A, Plunkett D (2013). Conceptual ethics II. Philosophy Compass.

[CR12] Carnap, R. (1950/1962). *Logical foundations of probability*. University of Chicago Press. [Page numbering adheres to the page numbering of the 1962 version.]

[CR13] Carnap, R. (1963). P. F. Strawson on linguistics and metatheory. In: P. Schilpp (Ed.), *The Philosophy of Rudolf Carnap*. Open Court: 933–940.

[CR14] Carnap, R. (1988). *Meaning and necessity. A study in semantics and modal logic.* Midway Reprint. [First print 1947, enlarged edition 1956].

[CR15] Chomsky N (1965). Aspects of the theory of syntax.

[CR16] Christensen D (2004). Putting logic in its place.

[CR17] Chuard P, Southwood N (2009). Epistemic norms without voluntary control. Noûs.

[CR18] Cohen S (1984). Justification and truth. Philosophical Studies.

[CR19] Cordes M (2020). The constituents of an explication. Synthese.

[CR20] Cordes, M., & Siegwart, G. (2018). Explication. In: J. Fieser & B. Dowden (Eds.), *Internet Encyclopedia of philosophy*. https://www.iep.utm.edu/explicat/.

[CR21] Dutilh Novaes C (2020). Carnapian explication and ameliorative analysis: A systematic comparison. Synthese.

[CR22] Dutilh Novaes C, Reck E (2017). Carnapian explication, formalisms as cognitive tools, and the paradox of adequate formalization. Synthese.

[CR23] Eder, A. M. A. (2020). No commitment to the truth. *Synthese*. 10.1007/s11229-019-02528-8 [Online first]

[CR24] Eder, A. M. A, Lawler, I., & van Riel, R. (Eds.) (2020a). *S.I. Philosophical Methods. Synthese, 197*.

[CR25] Eder AMA, Lawler I, van Riel R (2020). Philosophical methods under scrutiny: Introduction to the special issue philosophical methods. Synthese.

[CR26] Fitelson B, Hawthorne J, Eells E, Fetzer J (2010). How Bayesian confirmation theory handles the paradox of the ravens. The place of probability in science.

[CR27] Frege, G. (1884). *Die Grundlagen der Arithmetik – Eine logisch mathematische Untersuchung über den Begriff der Zahl.* Verlag von Wilhelm Koebner.

[CR28] Goldman A (1980). The internalist conception of justification. Midwest Studies in Philosophy.

[CR29] Graham PJ, Lackey J, Sosa E (2006). Liberal fundamentalism and its rivals. The epistemology of testimony.

[CR30] Greco J, Steup M, Sosa E (2008). Justification is not internal. Contemporary debates in epistemology.

[CR31] Greco J (2010). Achieving knowledge. A virtue-theoretic account of epistemic normativity.

[CR32] Hahn, S. (2013). *Rationalität: Eine Kartierung*. mentis.

[CR33] Hanna JF (1968). An explication of ‘Explication’. Philosophy of Science.

[CR34] Hawthorne J, Stanley J (2008). Knowledge and action. Journal of Philosophy.

[CR35] Hempel CG (1950). Towards a theory of interpretation and preciseness by Arne Næs. The Journal of Symbolic Logic.

[CR36] Hempel, C. G. (1952). *Fundamentals of concept formation in empirical science. (No. 7 of the International Encyclopedia of Unified Science)*. In O. Neurath, R. Carnap, & C. Morris (Eds.). Chicago: The University of Chicago Press. 10.1126/science.86.2235.400 17832642

[CR37] Justus J (2012). Carnap on concept determination: Methodology for philosophy of science. European Journal for Philosophy of Science.

[CR38] Kelly T (2003). Epistemic rationality as instrumental rationality: A critique. Philosophy and Phenomenological Research.

[CR39] Kitsik E (2020). Explication as a strategy for revisionary philosophy. Synthese.

[CR40] Langford, C. H. (1942). The notion of analysis in Moore’s philosophy. In P. Schilpp (Ed.), *The philosophy of G.E. Moore* (Open Court: pp. 319–342).

[CR41] Lehrer K (1990). Theory of knowledge.

[CR42] Maher P (2007). Explication defended. Studia Logica.

[CR43] Olsson EJ (2015). Gettier and the method of explication: A 60 year old solution to a 50 year old problem. Philosophical Studies.

[CR44] Olsson, E. J. (2017). Explicationist epistemology and epistemic pluralism. In A. Coliva & N. J. L. Linding Pedersen (Eds.), *Epistemic pluralism* (pp. 23–46). Palgrave Macmillan.

[CR45] Pinder M (2020). On Strawson’s critique of explication as a method in philosophy. Synthese.

[CR46] Pollock J (1987). Epistemic norms. Synthese.

[CR47] Pryor J, Steup M, Sosa E (2008). There is immediate justification. Contemporary debates in epistemology.

[CR48] Quine, W.v.O. (1960/2013). *Word and object*. MIT Press. [Page numbering adheres to the page numbering of the 2013 version.].

[CR49] Reck E, Wagner P (2012). Carnapian explication: A case study and critique. Carnap’s ideal of explication and naturalism.

[CR50] Schroeder M (2011). Ought, agents, and actions. Philosophical Review.

[CR51] Schupbach J (2017). Experimental explication. Philosophy and Phenomenological Research.

[CR52] Shepherd J, Justus J (2015). X-Phi and Carnapian explication. Erkenntnis.

[CR53] Spohn W (2012). The Laws of belief: Ranking theory and its philosophical Applications.

[CR54] Smithies D (2012). Moore’s paradox and the accessibility of justification. Philosophy and Phenomenological Research.

[CR55] Steup, M. (2018). Epistemology. In E. N. Zalta (Ed.). *The Stanford encyclopedia of philosophy* (Winter 2018 Edition). https://plato.stanford.edu/archives/win2018/entries/epistemology/.

[CR56] Strawson, P. F. (1963). Carnap’s views on constructed systems versus natural languages in analytic philosophy. In P. Schilpp (Ed.), *The philosophy of Rudolf Carnap* (Open Court: pp. 503–518).

[CR57] Strawson PF (1992). Analysis and metaphysics: An introduction to philosophy.

[CR58] Tarski A (1944). The semantic conception of truth. Philosophy and Phenomenological Research.

[CR59] Wedgwood R (2007). The nature of normativity.

[CR60] Wedgwood R (2012). Justified inference. Synthese.

[CR61] Wedgwood R, Ahlstrom-Vij K, Dunn J (2018). Epistemic teleology: Synchronic and diachronic. Epistemic consequentialism.

[CR62] White, R. (2013). Evidence cannot be permissive. In M. Steup, J. Turri, & E. Sosa (Eds.) (pp. 312–323). Hoboken: Wiley Blackwell.

[CR63] Wittgenstein, L. (1986). *Philosophical investigations*. Basil Blackwell [3rd edition; translated by G.E.M. Anscombe].

